# A new species of *Pseudosinella* Schäffer, 1897 (Collembola, Entomobryidae) from Hungary and Romania, with redescription of the related species *Pseudosinellahuetheri* Stomp, 1971

**DOI:** 10.3897/zookeys.1063.73094

**Published:** 2021-10-20

**Authors:** Daniel Winkler, Márton Tamás Németh, Cristina Fiera

**Affiliations:** 1 University of Sopron, Faculty of Forestry, Institute of Wildlife Management and Wildlife Biology, Bajcsy–Zs. str. 4, H–9400 Sopron, Hungary; 2 University of Sopron, Forest Research Institute, Paprét 17., H–9400 Sopron, Hungary; 3 Institute of Biology Bucharest of Romanian Academy, 296 Splaiul Independenţei, P.O. Box 56–53, 060031, Bucharest, Romania

**Keywords:** Chaetotaxy, *Pseudosinellahartnerae* sp. nov., springtails, taxonomy

## Abstract

A new species of the genus *Pseudosinella* Schäffer, 1897 from Hungary and Romania is described and illustrated. *Pseudosinellahartnerae***sp. nov.** belongs to the group with 5+5 eyes, and can be identified by its unique labial chaetotaxy (M_1_m_2_rel_1_L_2_) within this group. *Pseudosinellahuetheri* Stomp, 1971, the closest related species sharing the same dorsal macrochaetae formula (R_0_R_1_R_2_001/00/0101+2), is also redescribed here based on the holotype. Comparative analysis between the two species and among other related species is provided.

## Introduction

*Pseudosinella* Schäffer, 1897 is the largest collembolan genus, represented by 382 described species worldwide (Bellinger et al. 2021), inhabiting a wide range of habitats from xerophilic areas (e.g., Traser et al. 2006) through caves (e.g., Gisin and Gama 1969; Cipola et al. 2020) to wetlands like alluvial forests (e.g., Buşmachiu et al. 2017; Winkler and Mateos 2018).

As a part of soil biodiversity surveys in Hungary, the Bátorliget Pasture Nature Conservation Area was revisited in 2017. Among the collected Collembola material, a new species of *Pseudosinella* Schäffer, 1897 was discovered and is described in the present paper. Around the same time, Collembola samplings were carried out in Romania Mohoş Nature Reserve, from where the same *Pseudosinella* species was also found. The genus was previously represented by 17 species in Hungary (Dányi and Traser 2008; Winkler and Mateos 2018) and 24 species in Romania (Fiera 2013). However, its richness is probably underestimated and doubtless destined to increase with further taxonomic efforts in both countries.

The new species belongs to the group of species with 5+5 eyes. With the help of the computer assisted Delta identification key using the combination of chaetotaxic and other characters, originally designed by Christiansen et al. (1990) and regularly updated by Jordana et al. (2021), *Pseudosinellahuetheri* Stomp, 1971 was found to be its closest related species. Therefore, on this occasion, *Pseudosinellahuetheri* was also examined and redescribed from the holotype preserved in the Natural History Museum of Geneva (Switzerland).

## Material and methods

In October 2017, soil samplings were carried out in the Bátorliget Pasture Nature Conservation Area (East Hungary). A month later, soil mesofauna samplings were carried out also in Romania, around St. Ana Lake (Mohoş Nature Reserve). Springtails were extracted from the hand collected litter and soil samples within 14 days using a modified Berlese–Tullgren apparatus (without light or heating devices). The specimens were cleared using Nesbitt fluid and then mounted on permanent slides in Hoyer’s medium. The slides were examined under a Leica DM2500 LED microscope with conventional bright light and phase contrast.

Abbreviations used in text and figures are:

**Abd** abdominal tergite;

**accp** accessorial p–sensilla;

**Ant** antennal segment;

**a.s.l.** above sea level;

**HNHM**Hungarian Natural History Museum, Budapest;

**IBB**Institute of Biology Bucharest, Romanian Academy;

**NHMG** Natural History Museum of Geneva;

**Mac** macrochaeta;

**mic** microchaeta;

**psp** pseudopore;

**Th** thoracic tergite.

Symbols used in figures:

**open circle**Mac;

**black dots**mic;

**x** trichobotria.

### Terminology

Dorsal head chaetotaxy follows Gisin (1967), Jordana and Baquero (2007) and also the “AMS” nomenclature of Soto-Adames (2010). Clypeal chaetotaxy follows Yoshii and Suhardjono (1992). For the labial palp, the notation of Fjellberg (1999) was used. For labial chaetotaxy, Gisin’s nomenclature (1964) was applied, while for postlabial chaetotaxy, the notations of Chen and Christiansen (1993) and Cipola et al. (2018) were used. Dorsal chaetotaxy schemes of thoracic and abdominal segments follow Gisin (1967) and Szeptycki (1972, 1979), except for chaeta m_7_a on Abd III (following Wang et al. 2003) and chaeta p_8_p on Abd III (following Mateos 2008). The tergal specialized chaetae (S–chaetae) pattern follows Zhang and Deharveng (2015).

## Taxonomy

### Class Collembola Lubbock, 1873

**Order Entomobryomorpha Börner, 1913**

**Family Entomobryidae Schäffer, 1896**

**Subfamily Lepidocyrtinae Wahlgren, 1906**

#### Genus *Pseudosinella* Schäffer, 1897

##### Pseudosinella hartnerae

Taxon classificationAnimaliaEntomobryomorphaEntomobryidae

Winkler & Fiera sp. nov.

D61A6B94-1D17-5392-93A7-3C5A1E54792C

http://zoobank.org/EC5FA909-3CA5-4164-AAB2-E0AAFC5D989C

**Figure 1.**
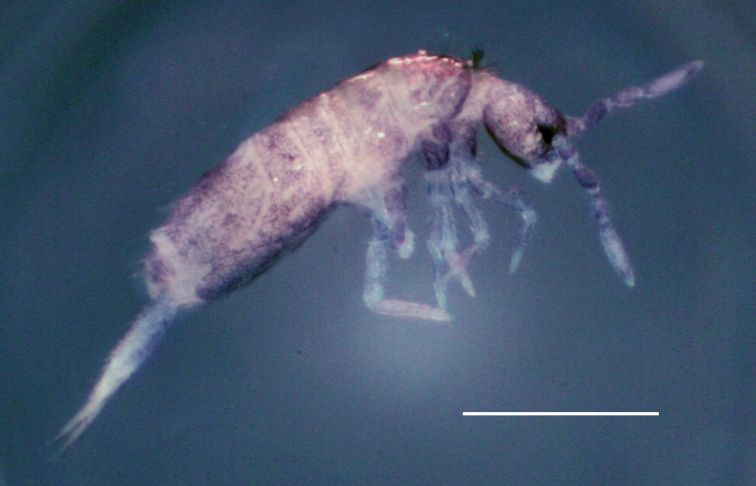
*Pseudosinellahartnerae* sp. nov. Habitus. Scale bar: 0.5 mm.

**Figure 2.**
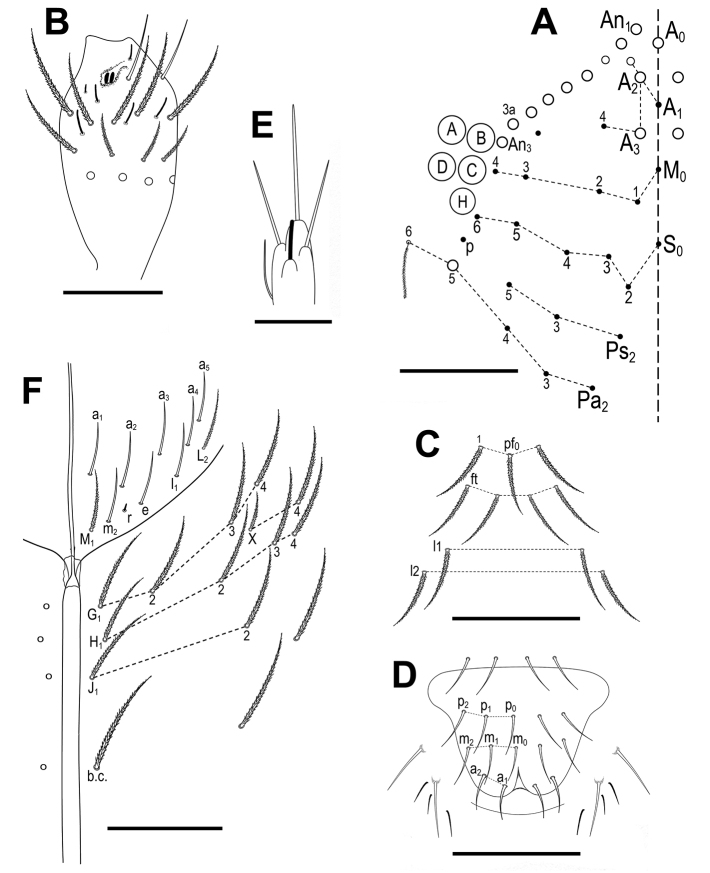
*Pseudosinellahartnerae* sp. nov. **A** head, dorsal chaetotaxy (left side) **B** antennae, Ant III with sensillar organ **C** clypeus, clypeal chaetae **D** labrum **E** labial papilla E with lateral process **F** labial triangle (right side) and ventral cephalic groove with labial and postlabial chaetotaxy. Scale bars: 0.05 mm (**A**); 0.03 mm (**B–D, F**); 0.01 mm (**E**).

**Figure 3.**
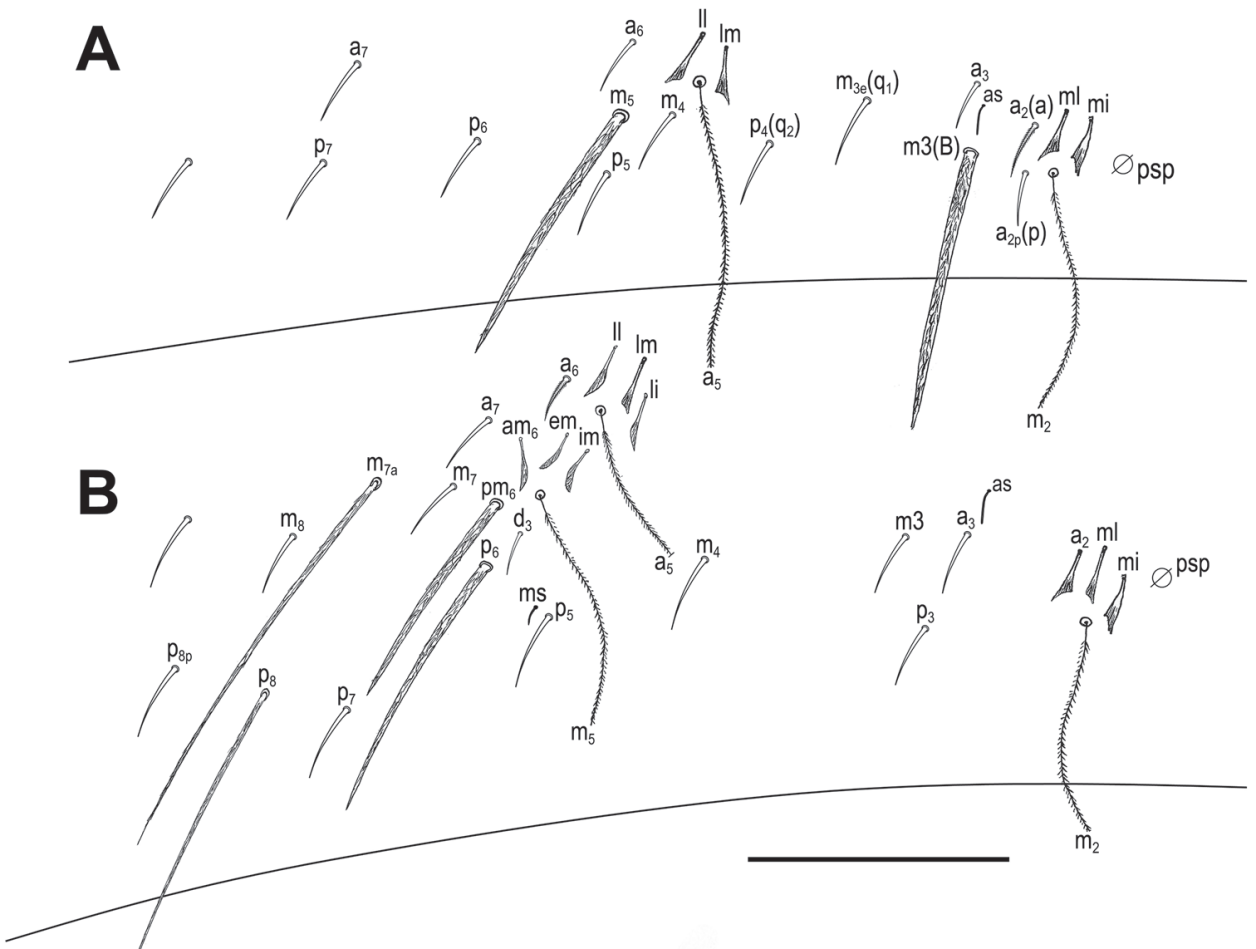
*Pseudosinellahartnerae* sp. nov. Abdomen dorsal chaetotaxy: **A**Abd II (left side) **B**Abd III (left side). Scale bar: 0.03 mm (**A, B**).

**Figure 4.**
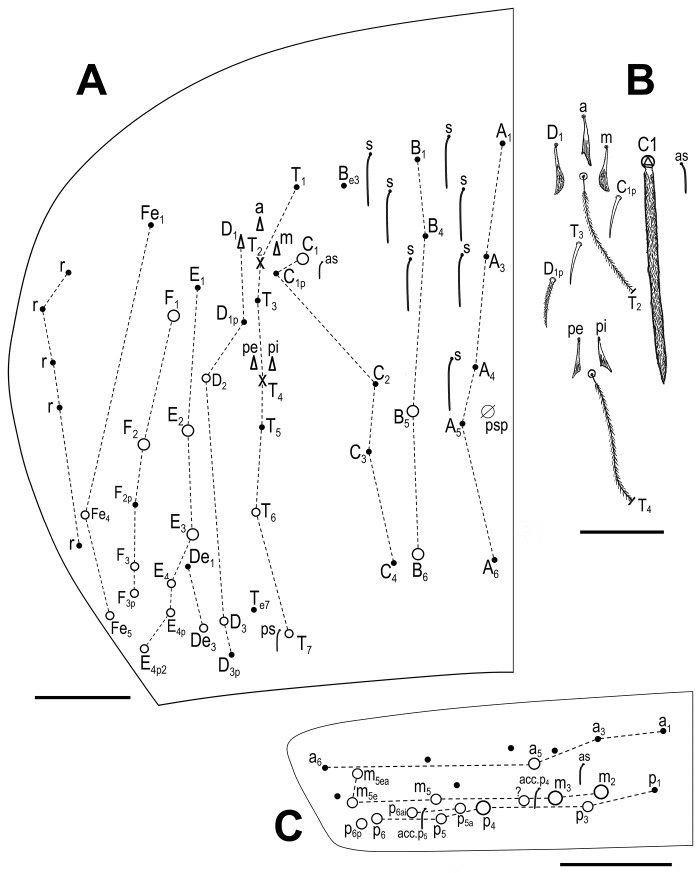
*Pseudosinellahartnerae* sp. nov. Abdomen dorsal chaetotaxy: **A**Abd IV (left side) **B**Abd IV trichobothrial complex (left side) **C**Abd V. Scale bars: 0.05 mm (**A**); 0.02 mm (**B**); 0.03 mm (**C**).

**Figure 5.**
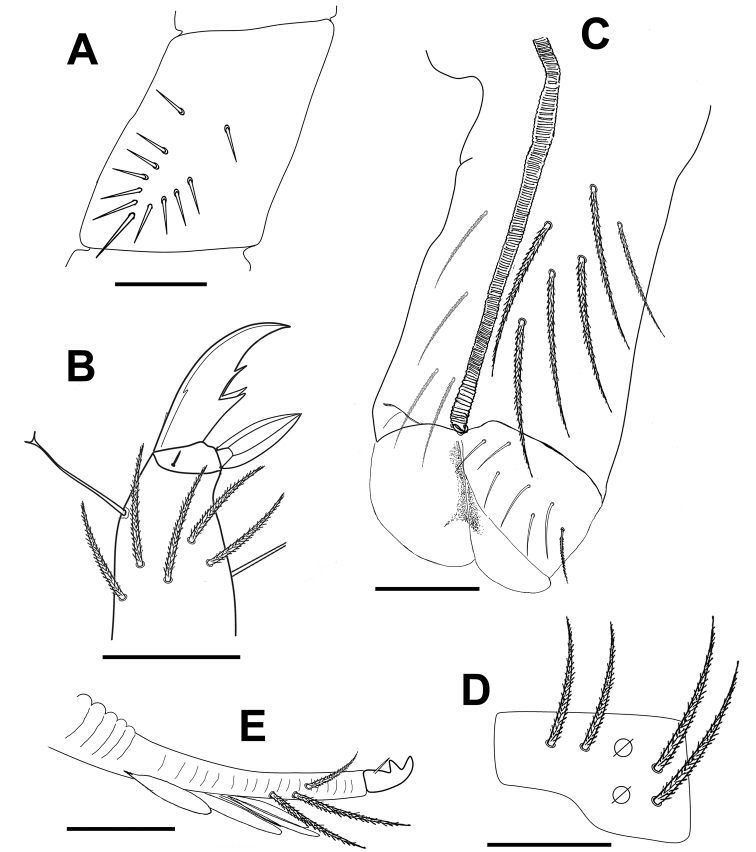
*Pseudosinellahartnerae* sp. nov. **A** trochanteral organ **B** leg III unguis and unguiculus **C** ventral tube anterior view (right side) and posterior view (left side) **D** manubrial plate **E** mucro and apical part of dens. Scale bars: 0.02 mm (**A, B, D, E**); 0.03 mm (**C**).

Figures 1 , 2 , 3 , 4 , 5

###### Type material.

***Holotype*:** Hungary. ♀ on slide (Nr. HNHM collpr-896), Bátorliget, Szabolcs–Szatmár–Bereg county, 161 m a.s.l., 47°46'11"N, 22°16'19"E, from litter, hand collecting, 8 Oct. 2017, leg. D. Winkler. ***Paratypes***: Two ♀ and one ♂ on slide (Nr. HNHM collpr-896); two ♀ and one specimen with sex not visible (Nr.: HNHM collpr-897); two ♀ (Nr. WD–coll–113 and WD–coll–114); same data as holotype. The holotype and four paratypes are deposited in the Hungarian Natural History Museum (HNHM), Budapest. Two paratypes are preserved in the first author’s collection at the University of Sopron, Faculty of Forestry, Sopron, Hungary; one paratype is kept in C. Fiera’s collection (IBB).

###### Other material.

Romania. Seven specimens (one ♂ on slide, Nr.: IBB Coll-12544; and six specimens of sex not determined in ethyl alcohol, vial Nr. IBB–26), Lake Saint Ana, Harghita County, Romania, 984 m a.s.l., 46°7'33"N, 25°53'28"E, 2 Nov. 2017, mixed forest with beech and fir, from litter, hand collecting, leg. C. Fiera.; 4 specimens (one ♀ on slide, Nr. IBB Coll–12545; and three specimens of sex not determined in ethyl alcohol, vial Nr. IBB–26), Mohoş peat bog, 1050 m a.s.l., 46°8'6"N, 25°53'59"E, 2 Nov. 2017, Scots pine forest, from peat moss *Sphagnum*, hand collecting, leg. C. Fiera. Preserved in the last author’s collection (IBB).

###### Diagnosis.

5+5 ocelli. Colour bluish-grey. Labial chaetotaxy M_1_m_2_rel_1_L_2_, r vestigial. Dorsal macrochaetae formula R_0_R_1_R_2_001/00/0101+2. Abd II chaetotaxy: paBq_1_q_2._Abd IV accessory chaeta s, anteriorly to trichobothrial complex, absent. Antennae and legs without scales. Unguis inner side with two paired basal teeth and one unpaired tooth. Unguiculus outer lamella smooth.

###### Description.

***Habitus*** (Fig. 1). Body length (without head and furca) 1.01–1.27 mm (holotype: 1.08 mm). Colour: Head, antennae, trunk and legs bluish-grey, blue shades also on manubrium and ventral tube. Eye patches dark blue.

***Head*.** With 5+5 eyes (ABCDH, with H only slightly smaller). Dorsal cephalic main macrochaetae formula R_0_R_1_R_2_P (according to AMS notation A_0_, A_2_, A_3_ and Pa_5_) (Fig. 2A). Maximum number of macrochaetae “An” on head 9+9. Antennal length to head diagonal length (measured from cervical edge to apex of labrum) ratio 1.2–1.4 (holotype: 1.3). Relation of antennal segments I–IV as 1 : 1.7 : 1.5 : 2.9 (holotype). Ant III sensillary organ composed of two rod-like sensilla partially behind a cuticular fold, guarded by three sensilla, one of them short, with spiny morphology (Fig. 2B). Ant IV without apical bulb. Clypeus with eleven subequal ciliated chaetae (three in row pf, four in row ft, two in row l1 and two in row l2) (Fig. 2C). Arrangement of chaetae on labrum 4/554, prelabral chaetae smooth; first (p), second (m) and apical (a) rows of labral chaetae also smooth, chaetae of p and m series about the same in size, not enlarged, a_1_–a_2_ thicker but not enlarged; labral edge with no differentiated papillae (Fig. 2D). Outer maxillary palp with two smooth chaetae and three smooth main sublobal hairs. Lateral process (*sensu*Fjellberg 1999) on papilla E finger-shaped, barely reaching the top of papilla (Fig. 2E). Labial anterior row with five smooth chaetae (a_1_–a_5_); formula of basal row M_1_m_2_rel_1_L_2_ with M_1_ and L_2_ ciliated and all other chaetae smooth (Fig. 2F). Chaeta r short (ratio of r/m_2_ 0.2–0.3). Ventral chaetotaxy with about 15 ciliate chaetae, postlabial formula 4 (G1–4), 2 (X, X4), 4 (H1–4), and 2 (J1–2) chaetae; b.c. present (Fig. 2F).

***Body*.** Body dorsal macrochaetae from Th II to Abd IV 00/0101+2. Mesothorax without macrochaetae. Two anterolateral S-chaetae (al and ms) present. Th III without Mac, anterolateral sensillum al present. Abd I with lateral S-microchaeta (ms). Chaetotaxy of Abd II–III as in Fig. 3A, B. Abd II chaetotaxy between two dorso-medial trichobothria paBq_1_q_2_ using Gisin’s symbols (Gisin 1967); following Szeptycki’s (1979) notation p=a_2_p, a=a_2_, B=m_3_, q_1_=m_3_e and q_2_=p_4_. Chaeta a as ciliated mic. Abd III chaeta d_3_ present. Chaetotaxy and trichobothrial complex on Abd IV as in Fig. 4A, B. Mac B_5_, B_6_, C_1_, E_2_, E_3_, F_1_ and F_2_ broader with broad sockets, D_2_, D_3_, De_3_, E_4_, E_4_p, E_4_p_2_, F_3_, F_3_p, Fe_4_, Fe_5_, T_6_ and T_7_ thinner with smaller sockets. Abd IV with five fan-shaped chaetae (D_1_, a, m, p_e_ and p_i_) associated with two trichobothria. Accessory chaeta s, associated with trichobotrium T_2_, absent. Nine S-chaetae (as, ps, and seven long dorsal S-chaetae) present. Dorsal chaetotaxy of Abd V as in Fig. 4C. Three S-chaetae (as, acc.p4 and acc.p5) typical for *Pseudosinella* present. Legs without scales. Trochanteral organ with up to 12 smooth spiny chaetae forming a V-shaped pattern (Fig. 5A). Unguis and unguiculus as in Fig. 5B. Unguis with subequal paired basal teeth at 35% from inner edge, and with a median unpaired inner tooth at 60% from inner edge, apical tooth absent. Outer side with paired lateral teeth and a short external tooth. Unguiculus lanceolate, external lamella smooth. Tibiotarsal tenent hair clavate, supraempodial chaeta on tibiotarsus III smooth and acuminate. Ratio of supraempodial chaeta / unguiculus around 0.9. Ventral tube without scales; with 6+6 subequal ciliated chaetae on anterior side and 4+4 subequal ciliated chaetae on posterior side; lateral flap with 1 ciliated and a maximum of 6 smooth chaetae (Fig. 5C). Manubrium ventrally with scales and 2+2 terminal ciliated chaetae. Manubrial plate with 2 larger inner chaetae and 2 chaetae external to the 2 pseudopores (Fig. 5D). Length of not ringed terminal dens about 4 times the length of mucro. Mucro with distal tooth equal to anteapical one; basal spine reaching tip of anteapical tooth (Fig. 5E). Ratio manubrium/dens/mucro as 16:15:1.

###### Ecology and distribution.

The type locality (Bátorliget Pasture Nature Conservation Area, Szabolcs–Szatmár–Bereg county, Hungary) of *Pseudosinellahartnerae* sp. nov. is a special relict mire and forest area with high biodiversity. The new species was collected from the upper layer and litter of a forest clearing with pioneer vegetation including silver birch (*Betulapendula*) trees. Specimens in Romania were collected from litter in the surrounding forest of the volcanic lake Saint Ana, and from peat mosses in the nearby Mohoș bog. Both Romanian sites are located in the Mohoş Nature Reserve, Harghita County. This new *Pseudosinella* is a phytodetriticolous, bryophilous and hygrophilous species.

###### Etymology.

The name of the new species is dedicated to former zoologist colleague and friend Dr. Anna Fenyősiné Hartner (1965–2006), an excellent specialist in myrmecology.

##### Pseudosinella huetheri

Taxon classificationAnimaliaEntomobryomorphaEntomobryidae

Stomp, 1971

C4A7CD6B-7FCB-5B01-A19B-B68E266C10A4

**Figure 6.**
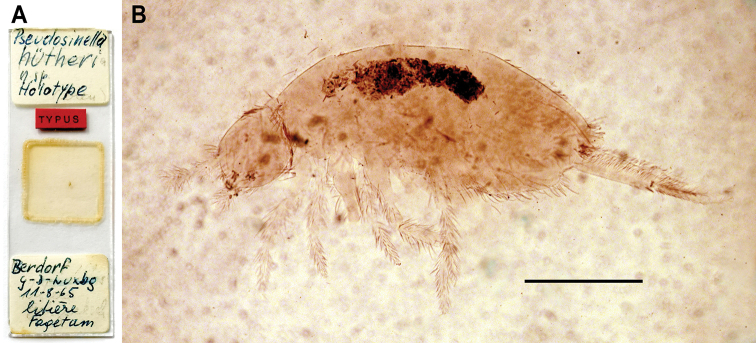
*Pseudosinellahuetheri* Stomp, 1971. **A** Photograph of the slide of the holotype from the NHMG Switzerland) **B** Habitus, holotype. Scale bar: 0.05 mm (**B**).

**Figure 7.**
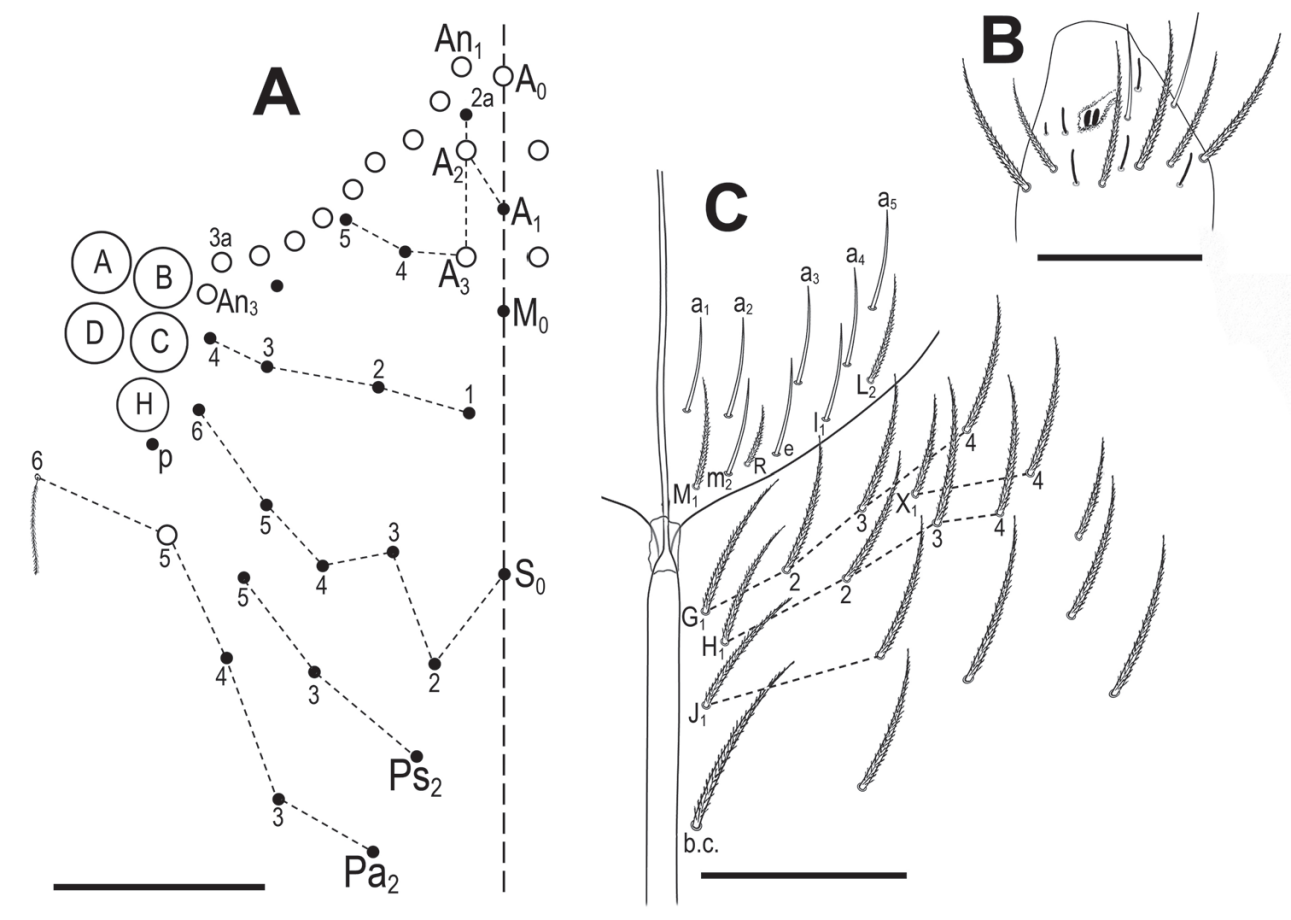
*Pseudosinellahuetheri* Stomp, 1971 **A** head, dorsal chaetotaxy (left side) **B** antennae, apex of Ant III with sensillar organ **C** labial triangle (right side) and ventral cephalic groove with labial and postlabial chaetotaxy. Scale bars: 0.05 mm (**A**); 0.03 mm (**B, C**).

**Figure 8.**
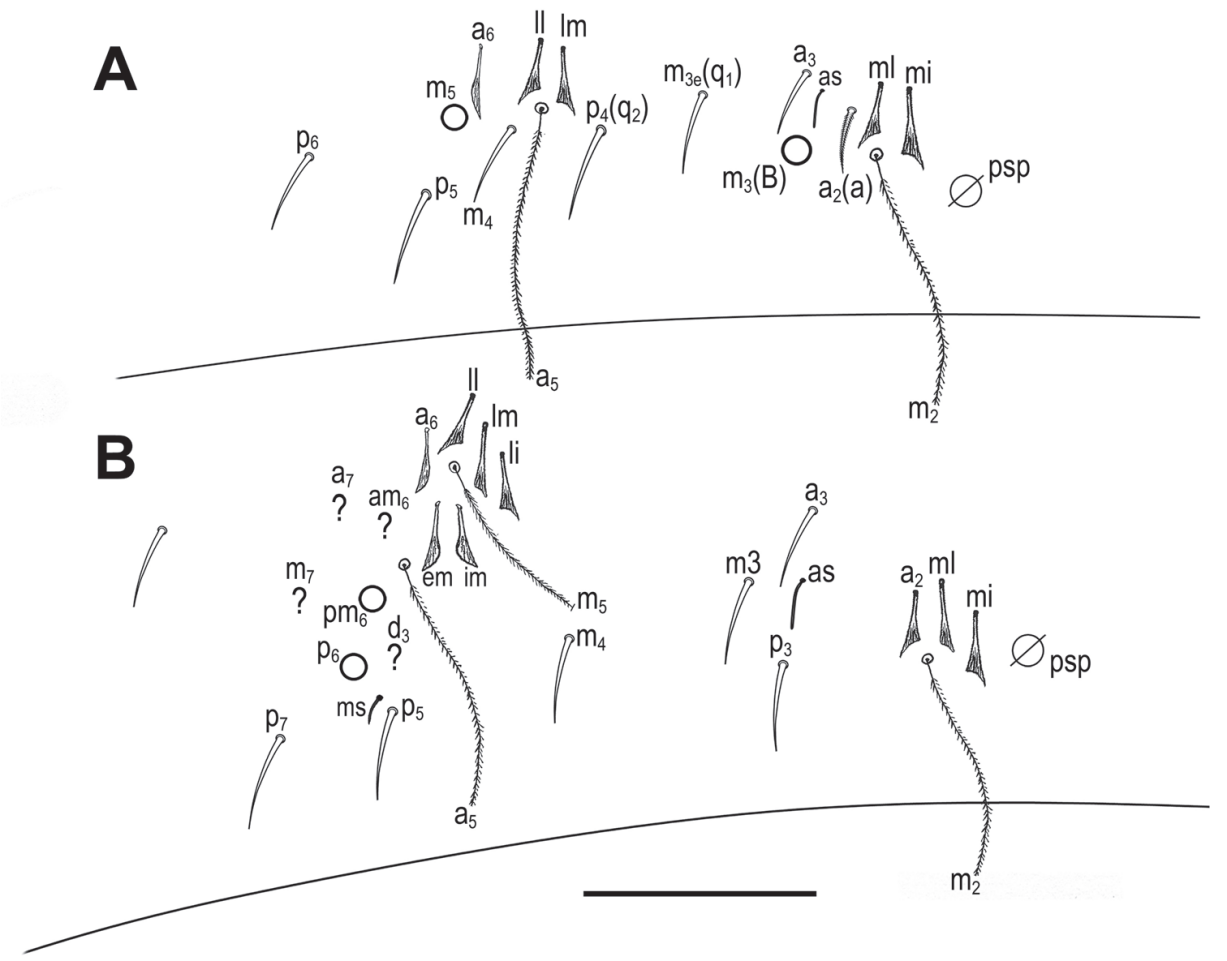
*Pseudosinellahuetheri* Stomp, 1971. Abdomen dorsal chaetotaxy: **A**Abd II (left side) **B**Abd III (left side). ? indicates the estimated position of chaetae that were not observed in the holotype due to the condition of the slide, but their presence is assumed. Scale bar: 0.05 mm (**A, B**).

**Figure 9.**
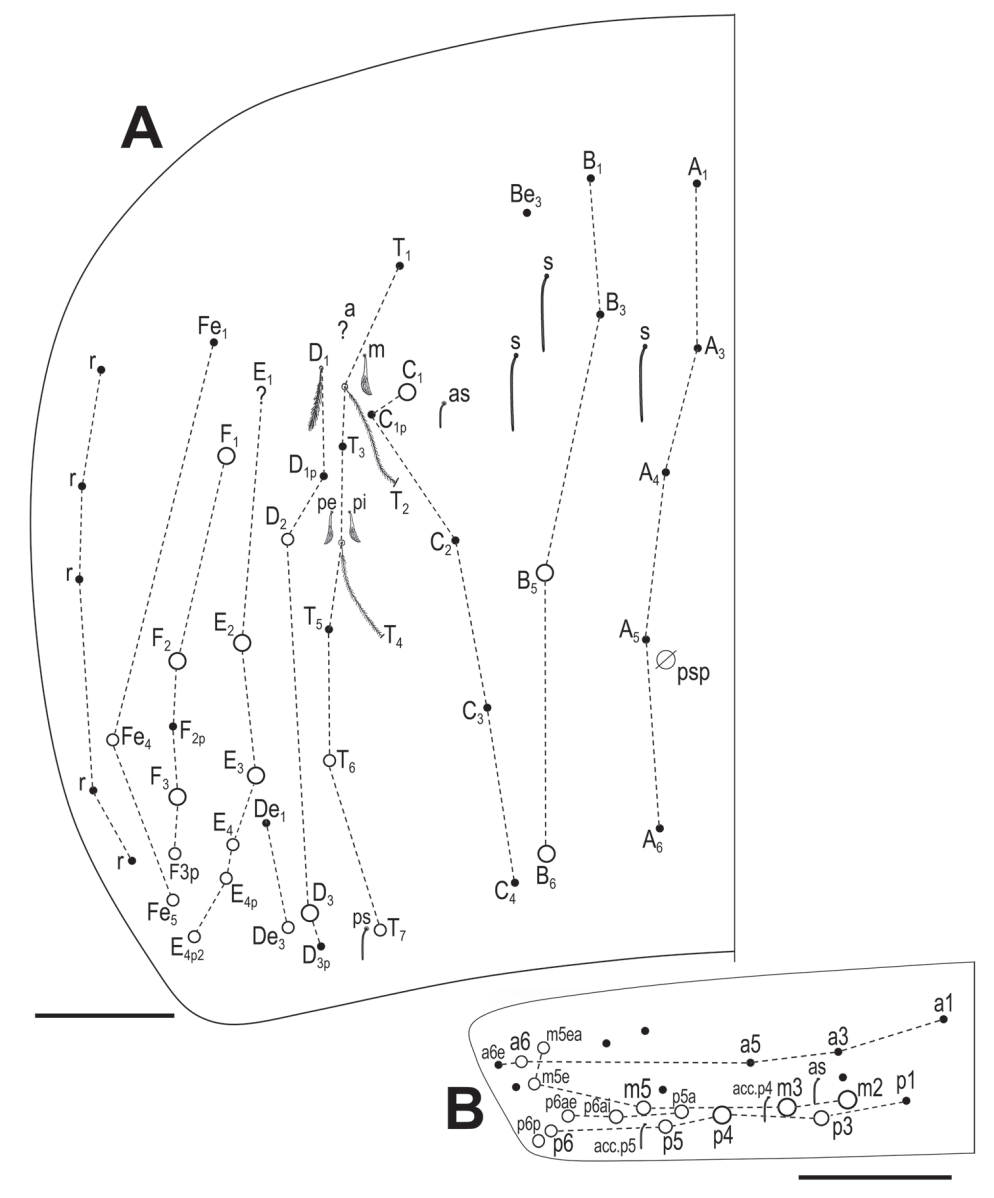
*Pseudosinellahuetheri* Stomp, 1971. Abdomen dorsal chaetotaxy: **A**Abd IV (left side) **B**Abd V. ? indicates the estimated position of chaetae that were not observed in the holotype due to the condition of the slide, but their presence is assumed. Scale bars: 0.05 mm (**A, B**).

**Figure 10.**
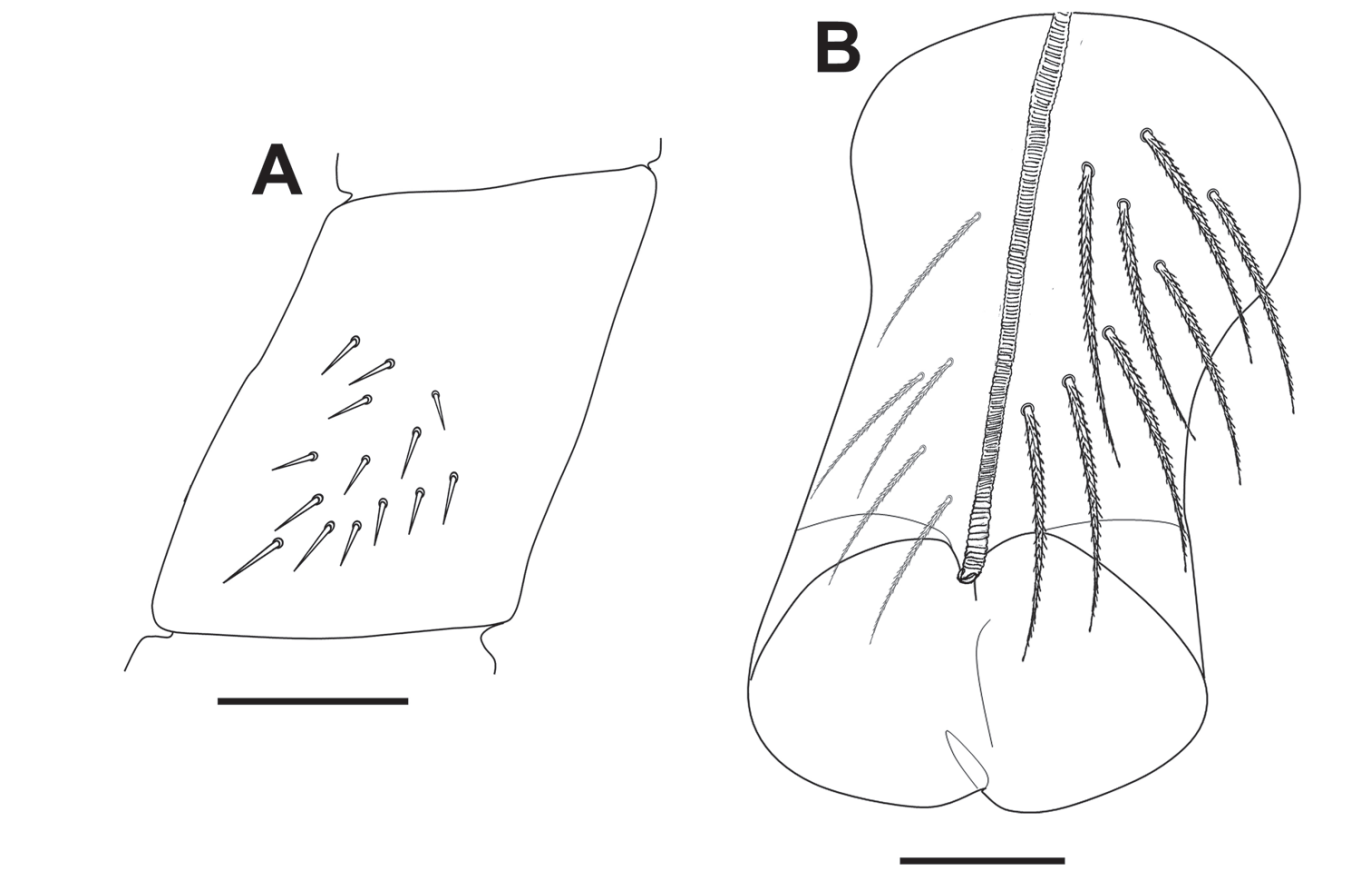
*Pseudosinellahuetheri* Stomp, 1971. **A** trochanteral organ **B** ventral tube anterior view (right side) and posterior view (left side). Scale bars: 0.03 mm (**A, B**).

Figures 6 , 7 , 8 , 9 , 10

###### Material examined.

***Holotype*:** Luxembourg. sex not visible, on slide, preserved in NHMG (Fig. 6A), Berdorf, “Zigzagschloeff” rocks, 357 m a.s.l., 49°49'33"N, 6°20'21"E, beech forest (*Fagetum*), from litter, 11.Aug.1965, leg. N. Stomp.

###### Diagnosis.

5+5 ocelli. Colour yellowish white. Labial chaetotaxy M_1_m_2_Rel_1_L_2_, R ~ 0.5 of M. Dorsal macrochaetae formula R_0_R_1_R_2_001/00/0101+2. Abdominal tergite II chaetotaxy: –aBq_1_q_2._Abd IV accessory chaeta s, anteriorly to trichobothrial complex, absent. Antennae and legs without scales. Unguis inner side with two paired basal teeth and one unpaired tooth, unguiculus outer lamella smooth.

###### Redescription.

***Habitus*** (Fig. 6B). Body length (without head and furca) 1.2–1.5 mm (Stomp 1971), holotype length 1.46 mm. Colour (after Stomp 1971): yellowish white, without any trace of pigment, neither on tergites and coxae nor on antennae. A few spots of blue pigment distributed in small dots around eyes. Eye patches blue.

***Head*.** With 5+5 eyes (ABCDH, with H only slightly smaller) (Fig. 7A; see also fig. 2 in Stomp 1971). Dorsal cephalic main macrochaetae formula R_0_R_1_R_2_P (according to AMS notation A_0_, A_2_, A_3_ and Pa_5_). Number of macrochaetae “An” on head 10+10 (Fig. 7A). Antennal length to head diagonal length ratio 1.4 (head diagonal measured from cervical edge to apex of labrum). Relation of antennal segments I–IV as 1 : 1.7 : 1.5 : 3.0. Ant III sensillary organ composed of two rod-like sensilla partially behind a cuticular fold, guarded by three sensilla, one of them shorter, spine-like (Fig. B). Ant IV without apical bulb. Arrangement of chaetae on labrum 4/554; prelabral chaetae smooth, first (p), second (m) and apical (a) rows of labral chaetae also smooth, chaetae of p and m series about the same in size, not enlarged, a_1_–a_2_ thicker but not enlarged; labral edge with no differentiated papillae (as in Fig. 2D). Outer maxillary palp with two smooth chaetae and three smooth main sublobal hairs. Lateral process (*sensu*Fjellberg 1999) on papilla E finger-shaped, barely reaching the top of papilla (see Fig. 2E). Labial anterior row formed by 5 smooth chaetae (a1–a5); formula of basal row M_1_m_2_Rel_1_L_2_ with M_1_, R and L_2_ ciliated and all other chaetae smooth (Fig. 7C). Chaeta R reduced (ratio of R/m_2_ 0.5). Ventral postlabial chaetotaxy with about 18 ciliate chaetae, postlabial formula 4 (G1–4), 2 (X, X4), 4 (H1–4), and 2 (J1–2) chaetae; b.c. present (Fig. 7C).

***Body*.** Body dorsal macrochaetae from Th II to Abd IV 00/0101+2. Mesothorax without Mac. Two anterolateral S-chaetae (al and ms) present. Th III without Mac, anterolateral sensillum al present. Abd I with lateral S-microchaeta (ms). Chaetotaxy of Abd II–III as in Fig. 8A, B. Abd II chaetotaxy between two dorso-medial trichobothria –aBq_1_q_2_ using Gisin’s symbols (Gisin 1967); following Szeptycki’s (1979) notation a=a_2_, B=m_3_, q_1_=m_3_e and q_2_=p_4_. Chaeta a as ciliated mic. Abd IV chaetotaxy as in Fig. 9A. Mac B_5_, B_6_, C_1_, D_3_, E_2_, E_3_, F_1_, F_2_ and F_3_ broader with broad sockets, D_2_, De_3_, E_4_, E_4_p, E_4_p_2_, F_3_p, Fe_4_, Fe_5_, T_6_ and T_7_ thinner with smaller sockets. Chaeta E_1_ not visible, its morphology unknown. Abd IV chaetae associated with the two trichobotria (m, p_e_ and p_i_) fan-shaped (chaeta a not visible, but with great certainty also fan-shaped) (see also Hüther 1969). Accessory chaeta s, associated with trichobotrium T_2_, absent. Five S-chaetae (as, ps, and three long dorsal S-chaetae) present. Dorsal chaetotaxy of Abd V as in Fig. 9B. Three S-chaetae (as, acc.p4 and acc.p5) typical for the genus present. Legs without scales. Trochanteral organ with 14 smooth spiny chaetae forming a V-shaped pattern (Fig. 10A). Unguis and unguiculus as in fig. 3 in Stomp (1971). Unguis with subequal paired basal teeth at 45% from inner edge, and with a median unpaired inner tooth at 65% from inner edge, apical tooth absent. A short external tooth also present. Unguiculus lanceolate, external lamella smooth. Tibiotarsal tenent hair spatulate, supraempodial chaeta on tibiotarsus III smooth and acuminate. Ratio of supraempodial chaeta / unguiculus ~0.9. Ventral tube without scales; with 8+8 subequal ciliated chaetae on anterior side and 5+5 subequal ciliated chaetae on posterior side (Fig. 10B); lateral flap with 4 ciliated and 7 smooth chaetae. Manubrium ventrally with scales and 2+2 terminal ciliated chaetae. Manubrial plate with 2 inner chaetae and 2 chaetae external to the 2 pseudopores (as in Fig. 5D). Length of not ringed terminal dens about 4 times the length of mucro. Mucro with distal tooth equal to anteapical one; basal spine reaching tip of anteapical tooth (as in Fig. 5E). Ratio manubrium/dens/mucro as 12:13:1.

###### Ecology and distribution.

*Pseudosinellahuetheri* was described from Luxembourg, from a beech forest near Berdorf (Stomp 1971). The original description reported the species also from Germany and Switzerland. Later, the species was found in Austria (Bretschko and Christian 1989), France (Ponge 2004) and Slovakia (Raschmanová et al. 2008). Dányi et al. (2006) collected a species close to *P.huetheri* in Romania, listed as “Pseudosinellacf.huetheri”, but without any differential character being mentioned.

## Discussion

Considering the number of eyes (5+5) and the similar dorsal main macrochaetae distribution (R000/00/0101+2 or R001/00/0101+2), *Pseudosinellahartnerae* sp. nov. is close to *P.altamirensis* Baquero, Jordana, Labrada & Luque, 2020; *P.horaki* Rusek, 1985; *P.huetheri*; *P.mauli* Stomp, 1972; *P.mucronata* Gouze & Deharveng, 1987; and *P.sandelsorum* Gruia, 1977 (Table 1). The new species is, however, characterized by a unique basal labial chaetotaxy. While *P.horaki*, *P.mauli* and *P.sandelsorum* have M_1_, M_2_, E, L_1_ and L_2_ as ciliated chaetae, in *P.hartnerae* sp. nov. only M_1_ and L_2_ are ciliated, and m_2_, r, e, and l_1_ are smooth. *Pseudosinellaaltamirensis* bears two ciliated labial chaetae (M_1_ and R), and *P.mucronata* has R and occasionally also M_1_ as ciliated chaetae, while all other chaetae are smooth. Only *P.huetheri* shows a similar morphology of labial chaetae, with the exception of chaeta R present as a fairly developed ciliated chaeta (r smooth and reduced in *P.hartnerae* sp. nov). The new species differs from *P.huetheri* also by the colour pattern and by the presence of Abd II chaeta p (a_2p_). The new species differs from *P.altamirensis* and *P.mucronata* also by the presence of subocular cephalic marcrochaeta Pa_5_. The new species differs from *P.altamirensis* and *P.mauli* by the absence of accessory chaeta s on Abd IV. Besides, the new species differs from *P.sandelsorum* by the number of teeth of the inner unguis. *Pseudosinellahartnerae* sp. nov. and *P.huetheri* share the number of chaetae (2) external to the pseudopores on the manubrial plate, which is greater for *P.altamirensis* (7–12), *P.mauli* (3) and *P.sandelsorum* (4–10).

**Table 1.** Comparison of *P.hartnerae* sp. nov. with related species with 5+5 eyes and similar dorsal macrochaetae distribution.

Species	Ch1	Ch2	Ch3	Ch4	Ch5	Ch6	Ch7	Ch8	Ch9	Ch10	Ch11	Ch12
*P.altamirensis*	pale with blue pigmentation	M_1_m_2_Rel_1_l_2_	R000/00/0101+2	1.6–2.2	–	+	3	35%	40%	1	2+7–12	2
*P.horaki*	pale greyish blue	M_1_M_2_REL_1_L_2_	R001/00/0101+2	1.6	+	–	3	50%	75%	2	U	1
*P.huetheri*	yellowish white	M_1_m_2_Rel_1_L_2_	R001/00/0101+2	1.4	–	–	3	45%	65%	2	2+2	1
*P.mauli*	bluish black	M_1_M_2_rEL_1_L_2_	R001/00/0101+2	1.3	+	+	3	~45%	65%	2	2+3	1
*P.mucronata*	diffuse pigmentation	m_1_(M_1_)m_2_Rel_1_l_2_	R000/00/0101+2	1.8–2.0	–	–	3	~45%	60%	1	U	2
*P.sandelsorum*	dark blue pigment on Ant and legs	M_1_M_2_REL_1_L_2_	R001/00/0101+2	1.3	+	–	4	~50%	70%	2	2+4–10	1
*P.hartnerae* sp. nov	bluish-grey	M_1_m_2_rel_1_L_2_	R001/00/0101+2	1.2–1.4	+	–	3	35%	60%	2	2+2	1

Legend. **Ch1**: body colour. **Ch2**: basal labial chaetotaxy formula. **Ch3**: body dorsal macrochaetae formula. **Ch4**: antennal length to head diagonal length ratio. **Ch5**: Abd II chaeta **p**: (+) present or (–) absent. **Ch6**: Abd IV supplementary chaeta **s**: (+) present or (–) absent. **Ch7**: number of teeth of inner unguis. **Ch8**: distance of distal paired claw tooth from the base as a % of total claw length. **Ch9**: distance of distal unpaired claw tooth from the base as a % of total claw length. **Ch10**: tenent hair shape: (1) acuminate, (2) clavate. **Ch11**: number of inner and outer chaetae on manubrial plate. **Ch12**: habitat: (1) surface, (2) cave. “U”, unknown.

## Supplementary Material

XML Treatment for Pseudosinella hartnerae

XML Treatment for Pseudosinella huetheri
